# Moderating Effect of Cortical Thickness on BOLD Signal Variability Age-Related Changes

**DOI:** 10.3389/fnagi.2019.00046

**Published:** 2019-03-12

**Authors:** Daiana R. Pur, Roy A. Eagleson, Anik de Ribaupierre, Nathalie Mella, Sandrine de Ribaupierre

**Affiliations:** ^1^School of Biomedical Engineering, Western University, London, ON, Canada; ^2^Department of Electrical and Computer Engineering, Western University, London, ON, Canada; ^3^Department of Psychology, University of Geneva, Geneva, Switzerland; ^4^Department of Clinical Neurological Sciences, Schulich School of Medicine, Western University, London, ON, Canada

**Keywords:** signal variability, BOLD fMRI, structural alterations, cortical morphology, aging, cortical thickness, neural processing, biomarker

## Abstract

The time course of neuroanatomical structural and functional measures across the lifespan is commonly reported in association with aging. Blood oxygen-level dependent signal variability, estimated using the standard deviation of the signal, or “BOLD_SD_,” is an emerging metric of variability in neural processing, and has been shown to be positively correlated with cognitive flexibility. Generally, BOLD_SD_ is reported to decrease with aging, and is thought to reflect age-related cognitive decline. Additionally, it is well established that normative aging is associated with structural changes in brain regions, and that these predict functional decline in various cognitive domains. Nevertheless, the interaction between alterations in cortical morphology and BOLD_SD_ changes has not been modeled quantitatively. The objective of the current study was to investigate the influence of cortical morphology metrics [i.e., cortical thickness (CT), gray matter (GM) volume, and cortical area (CA)] on age-related BOLD_SD_ changes by treating these cortical morphology metrics as possible physiological confounds using linear mixed models. We studied these metrics in 28 healthy older subjects scanned twice at approximately 2.5 years interval. Results show that BOLD_SD_ is confounded by cortical morphology metrics. Respectively, changes in CT but not GM volume nor CA, show a significant interaction with BOLD_SD_ alterations. Our study highlights that CT changes should be considered when evaluating BOLD_SD_ alternations in the lifespan.

## Introduction

Normal aging is associated with marked functional and structural neuroanatomical alterations in cortical thickness (CT), gyrification, cortical surface area (CA), gray (GM), and white matter volume (WM) (Salat et al., 2009; Thambisetty et al., 2010; McGinnis et al., 2011; Hogstrom et al., 2013). Importantly, magnetic resonance imaging studies (MRI) show that the magnitude and rate of change of these cortical morphometry metrics is not constant across the cortex but rather it varies with age and brain region (Raz, 2005; Jiang et al., 2014; Storsve et al., 2014; Dotson et al., 2016), and is reported to accelerate with increasing age (Driscoll et al., 2009; Jiang et al., 2014). For example, a longitudinal study of alterations in cortical morphometry in older adults found accelerated changes with increasing age in temporal and occipital cortices (Storsve et al., 2014). Furthermore, other studies report that the temporal lobes are most vulnerable to age-related morphometric changes, and that these changes reflect age-related cognitive impairment (Singh et al., 2006; Fjell et al., 2009; Pacheco et al., 2015). There is considerable evidence that neuroanatomical alterations reflect underlying functional alterations, especially in cognition (Fjell et al., 2006; Rossini et al., 2007; Ziegler et al., 2010; Achiron et al., 2013). In fact, functional magnetic resonance imaging (fMRI) studies, which rely on the blood oxygen level-dependent (BOLD) signal as a correlate of neuronal activity, report that changes in cortical morphology across adult lifespan impact the hemodynamic properties of the brain. For example, there are cortical laminar differences in BOLD signal (Tian et al., 2010; Huber et al., 2015), thicker cortical regions were reported to have a lower relative oxygen extraction fraction (Zhao et al., 2016). Therefore, since aging is associated with significant neuroanatomical alterations, these should be considered when assessing function (i.e., cognitive ability) using the BOLD signal.

The Standard Deviation of the BOLD signal can be used to estimate variability (hereafter, “BOLD_SD_”), and is believed to reflect the brain’s dynamic ability to undergo fast moment-to-moment switching through network reconfigurations (Garrett et al., 2010, 2014; Grady and Garrett, 2014). It is an emerging index of cognitive health in aging, with higher regional BOLD_SD_ being associated with enhanced performance on certain cognitive tasks (i.e., task switching) but not on others (i.e., distractor inhibition) (Armbruster-Genc et al., 2016; Guitart-Masip et al., 2016). Generally, increased BOLD_SD_ is associated with younger age, faster and more consistent performance on cognitive tasks, and cognitive flexibility (Garrett et al., 2013a; Armbruster-Genc et al., 2016). The physiological mechanisms underlying BOLD_SD_ remain largely unknown. For instance, decreased dopaminergic transmission is proposed to be associated with decreased BOLD_SD_ in subcortical areas in older adults compared to younger ones (Guitart-Masip et al., 2016). Another study reported that higher BOLD_SD_ is associated with superior pain coping capabilities (Rogachov et al., 2016). However, there are few studies investigating the interaction between age-related alterations in cortical morphology and BOLD_SD_. One study reported that increased microstructural integrity of WM pathways is associated with greater BOLD_SD_ (Burzynska et al., 2015). Given that neuroanatomical alterations associated with normative aging are known to influence cognitive performance, and that they influence the BOLD signal, their impact as physiological confounds to BOLD_SD_ should be investigated. This is particularly relevant because there is a considerable degree of inconsistency of methods used and results across studies investigating BOLD_SD_. In fact, some studies report greater regional BOLD_SD_ in older adults (Garrett et al., 2010, 2011; Nomi et al., 2017), individuals with stroke (Kielar et al., 2016), multiple sclerosis (Petracca et al., 2017), Alzheimer disease (Makedonov et al., 2013, 2016; Scarapicchia et al., 2018) and other neurological disorders (Zöller et al., 2017).

The objective of this study is to investigate the contribution of cortical morphology (i.e., CT, CA, and GM volume) to age-related BOLD_SD_ changes in older adults. A longitudinal framework, consisting of two scan points, should help reduce some of the inter-individual variance in neuroanatomy by accounting for external factors such as lifestyle, and various socio-economic and demographic factors. To this end, we investigated global and regional differences in BOLD_SD_ in a group of older adults scanned twice at an interval of approximately 2.5 years, and regressed the effect of cortical morphology by introducing CT, CA, and GM as covariates. We hypothesized that cortical morphology metrics show an interaction with BOLD_SD_. We predict that age-related neuroanatomical alterations in CT, CA, GM are physiological confounds to BOLD_SD_ measures, and that consequently adjusting for these metrics may help “unmask” the functional relevance of BOLD_SD_.

## Materials and Methods

### Participants

All data obtained for the present study were obtained from the longitudinal Geneva Aging Study, after approval by the ethics committee of the Faculty of Psychology and Educational Sciences of the University of Geneva and the Swiss Ethic committee. Older subjects were initially recruited either from the University of the Third Age of Geneva or through newspaper and association advertisements for pensioners, as part of a larger longitudinal study. All participants gave written informed consent and older adults received a small amount of money as a compensation for their transportation fees.

Our initial sample consisted of 31 older adults scanned twice (mean age at first scan = 71.65 ± 6.03 years, mean age at second scan = 74.06 ± 5.99 years; 9 males). These subjects were chosen, within our pool, because they were the only ones that had undergone two T1 structural images and task fMRI scans, as well as other cognitive tests. Participants were screened for health problems with a questionnaire. The structural MRIs were inspected to rule out severe abnormalities (white matter changes, ventricular enlargement, tumors etc.). Three of the participants showed signs of Parkinson or lesion on their anatomical MRI, so they were excluded from the final sample (*n* = 28). All models and results in this current paper thus reflect 28 older adults (mean age at first scan = 71.61 ± 6.21 years, mean age at second scan = 74.07 ± 6.15 years; 7 males). The scans were 2.46 ± 0.69 years apart.

### MRI Data Acquisition

Participants were scanned in a Siemens Trio 3T magnet. A BOLD fMRI task-rest sequence was administered using a reaction time paradigm, where the participant had to indicate on which side a cross was changing into a square, as fast as possible (Mella et al., 2013). The task consisted of eight experimental blocks, interspersed with eight resting blocks (respectively, 52–20 s). The BOLD activity was obtained using an echo planar imaging acquisition (echo time, TE = 30 ms, time repetition, TR = 2100 ms, flip angle = 80°, field of view, FOV = 205 mm). Then, a structural T1-weighted MRI was acquired (TE = 2.27 ms, TR = 1900 ms, FOV = 256 mm, voxel size 1.0 mm × 1.0 mm × 1.0 mm).

### Structural Data Processing

Structural T1-weighted MR images were analyzed using Freesurfer version 6.0, a widely used and freely available automated processing pipeline^1^, which allows surface-based three dimensional reconstruction and quantification of cortical morphology. The standard steps for analysis were implemented (using “recon-all” pipeline with the default set of parameters). Regional measures of GM, CT, and CA for each hemisphere were obtained using the automated anatomic parcellation procedure. Technical details are found in prior publications (Dale et al., 1999; Fischl et al., 1999a,b, 2001; Fischl and Dale, 2000; Zhang et al., 2001; Salat et al., 2004; Winkler et al., 2012). In brief, T1-weighted images underwent preprocessing steps including motion correction, brain extraction, intensity normalization, and Talairach transformation (Sled et al., 1998; Smith, 2002; Ségonne et al., 2004; Reuter et al., 2010). GM and WM surface boundaries were reconstructed to estimate the distance between them across the cortex (Fischl and Dale, 2000). The generated cortical models were inflated into spheres to be registered to a spherical atlas and parcellated into regions of interest using Destrieux atlas (Fischl and Dale, 2000; Segonne et al., 2007; Destrieux et al., 2010).

### Functional Data Processing

Processing of the functional data were performed using FSL version 5.0 (Analysis Group, FMRIB, Oxford, United Kingdom (Smith et al., 2004). Standard preprocessing were followed using FSL’s FEAT and FSL’s Melodic for functional data (Jenkinson et al., 2012). Briefly, for each participant preprocessing steps included motion correction, slice timing, spatial normalization, highpass temporal filtering (100 s), smoothing (kernel 5 mm FWHM), and linear registration (12 degrees of freedom) of the functional data to the high-resolution T1 structural image, and from T1 to 1 mm standard space (MNI 152). Additionally, FSL’s Melodic was used to correct each functional image for artifacts using automatic dimensionality estimation via independent component analysis (ICA) (Beckmann and Smith, 2004). Change in cortical morphology between the two scanning sessions was determined as cortical morphology metrics GM (delta.volume), CT (delta.thickness), CA (delta.area) at timepoint 2–at timepoint 1.

#### BOLD Signal Variability Calculation

As part of the Geneva Dataset, the subjects were performing different cognitive tasks, and for the current study, only the fixation/rest blocks from the block design task fMRI were selected to calculate BOLD_SD,_ as previously described (Garrett et al., 2010). BOLD_SD_ analysis was restricted to the GM using participant specific GM mask obtained from FSL’s FAST. First, fixation blocks were normalized so that the overall four-dimensional mean across brain and block was 100. Next, for each voxel, the block mean was subtracted to remove block-wise drift, followed by concatenation of all blocks. The standard deviation of the normalized mean of the concatenated fixation blocks was used to obtain BOLD_SD_ values for each brain region (*n* = 148) of each subject, as defined by the Destrieux Atlas (Destrieux et al., 2010), using in-house MATLAB code. Change (delta.variability) in variability between the two scanning sessions was calculated as variability at timepoint 2–variability at timepoint 1. BOLD_SD_ encompassing both timepoints was introduced as “variability” (see section “Regional Model”).

### Statistical Analysis

Linear mixed effects models (LMMs) were used to investigate the potential confounding effect of cortical morphology, GM, CT, CA on BOLD_SD_ (Bates, 2005; Zuur et al., 2011).The LMMs allow estimation of the effects of explanatory variables (“fixed effects”) and their interactions on the dependent variable (i.e., BOLD_SD_), while statistically controlling for the effects of randomly selected participants (“random effects”) on the dependent variable (BOLD_SD_). Multiple models were run and the likelihood-ratio test was used to (1) investigate if introducing subjects as random effects improves the fit of the model (2) to select the optimal combination of fixed effects fitted with maximum likelihood, while keeping the random effects structure the same. Therefore, the likelihood-ratio test via ANOVA was used to compare the goodness of fit of different models. R statistical software package (R Core Team, 2013)^2^ was used for all statistical analyses. Correlation between cortical morphology measures were computed using cor function in R. All models were fitted using the “lmer” or “lm” function in R. “lmerTest” R package was used to obtain summary table and *p*-values for linear mixed models via Satterthwaite’s degrees of freedom method (Kuznetsova et al., 2017). A spatio-temporal approach to LMMs allowed characterization of regionally specific variation across the brain (Bernal-Rusiel et al., 2013). This approach was implemented to investigate if there is a significant change in BOLD variability across time (1) all cortical regions (2) region specific. Random effect structure with subjects varying in their “baseline” BOLD variability was retained (random intercept, 1| ID). Additionally, to model a different rate of change in the expected response levels, time varying predictors were introduced by random slopes (i.e., thickness, time) (“Regional Model”). To reduce spatial correlation issues an LMM model with the described structure was applied at each spatial location (i.e., region of interest) independently. Each model produced a parameter which quantifies the mean change in BOLD variability (delta.variability) for that region. *P*-values were corrected for multiple comparisons using false discovery rate (FDR) at *q* = 0.05 (Benjamini and Yekutieli, 2001).

#### Models

Model 1 < lm (delta.variability∼ region).
Model 2 < - lmer [delta.variability∼ region+(1| ID), REML = F].
Model 3 < - lmer [delta.variability ∼ delta.thickness + region+(1| ID), REML = F].
Model 4 < - lmer [delta.variability ∼ delta.area + region + (1| ID), REML = F].
Model 5 < - lmer [delta.variability ∼ delta.thickness + delta.area + region+(1| ID), REML = F].
Model 6 < - delta.variability ∼ delta.volume + region + (1 | ID).

##### Final Model

lmer [delta.variability∼ delta.thickness + region+(1| ID)].

##### Regional Model

lmer [variability∼ time + thickness + (1+thickness + time| ID)].

## Results

### Relations Between Cortical Thickness and BOLD Signal Variability Age-Related Changes

The functional and structural data of 28 participants was assessed. As expected, our study showed that CA and GM are highly correlated *r* = 0.904, *p* < 0.001 (95% Cl 0.901–0.908) and consequently collinearity was suspected. Multiple LMMs were utilized to assess the effect of neuroanatomical metrics CT, CA, and GM on BOLD_SD_ age-related changes. Results from the linear mixed effect models run with likelihood-ratio test via ANOVA, are presented in Table 1. (A) indicates that subject intercept should be introduced as a random effect, while (B–E) show the steps that have led to the final model. Specifically, Table 1. (B,C) indicate that introducing CT or CA as covariates, separately, each significantly improve the fit of the model *p* < 0.0001, *p* < 0.05, respectively. However, from (D) it is apparent that adding CA to a model that already has CT as a covariate does not improve the fit of the model, meaning that CT only should be included in the final model (see section “Final Model”). (E) indicates that introducing GM as a covariate does not improve the fit of the model. Neither CT, GM, nor CA mean changes were significant as tested with LMMs. Regional Model LMM indicated that neither overall nor regionally specific mean BOLD_SD_ change was significant after FDR correction.

**Table 1:** Results from likelihood-ratio test via ANOVA for model comparison (fit with “lmer” function in R).

	Df	AIC	BIC	logLik	Deviance	Chisq Chi	Df.	*P*r > Chisq
**(A) Determine if random effects for subject intercept should be introduced**								

Model 1	Delta.variability ∼ Region							
Model 2	Delta.variability ∼ Region + (1| ID)							
Model 1	149	-2771.9	-1828.8	1534.9	-3069.9			
Model 2	150	-3744.8	-3744.8	2022.4	-4044.8	974.97	1	<2.2e-16

**(B) Determine if introducing thickness as a covariate improves the goodness-of-fit**		
Model 2	Delta.variability ∼ Region + (1| ID)		
Model 3	Delta.variability ∼ Delta.thickness + Region + (1| ID)		
Model 2	150	-3744.8	-2795.4	2022.4	-4044.8			
Model 3	151	-3765.5	-2809.8	2033.8	-4067.5	22.691	1	1.903e-06

**(C) Determine if introducing area as a covariate improves the goodness-of-fit**		
Model 2	Delta.variability ∼ Region + (1| ID)		
Model 4	Delta.variability ∼ Delta.area + Region + (1| ID)		
Model 2	150	-3744.8	-2795.4	2022.4	-4044.8			
Model 4	151	-3749.0	-2793.3	2025.5	-4051.0	6.2025	1	0.01276

**(D) Determine if introducing both area and thickness as covariates as compared to just one improves the goodness-of-fit**		
Model 3	Delta.variability ∼ Delta.thickness + Region + (1| ID)		
Model 5	Delta.variability ∼ Delta.thickness + Delta.area + Region + (1| ID)		
Model 3	151	-3765.5	-2809.8	2033.8	-4067.5			
Model 5	152	-3766.7	-2804.7	2035.4	-4070.7	3.2151	1	0.07296

**(E) Determine if introducing gray matter volume as covariate improves the goodness-of-fit**		
Model 2	Delta.variability ∼ Region + (1| ID)		
Model 6	Delta.variability ∼ Delta.volume + Region + (1| ID)		
Model 2	150	-3744.8	-2795.4	2022.4	-4044.8			
Model 6	151	-3743.1	-2787.3	2022.5	-4045.1	0.2245	1	0.6357

*AIC, akaike information criterion; BIC, Bayesian information criterion; Chisq Chi, chi-square test statistic; Df, degrees of freedom; logLik, log-likelihood; *P*r > Chisq. *P* < 0.05. ID represents subject identification number*.

## Discussion

### Cortical Thickness and Its Association With BOLD_SD_

In this study we aimed at determining the contribution of cortical morphology to BOLD_SD_ in order to better understand the physiological nature of brain function capture by BOLD_SD_. BOLD_SD_ changes across the lifespan have been shown to be robust to certain vascular factor such as cerebral blood flow, BOLD cerebrovascular reactivity, maximal BOLD signal change (Garrett et al., 2017), as well as GM volume changes (Nomi et al., 2017). However, using LMMs, we found that functional alterations in aging as captured by BOLD_SD_ are confounded by the structural metric CT. This is not surprising, considering that it is well known that BOLD signal change/activation is dependent on the laminar organization of the cortex, and consequently is influenced by its depth or thickness (Koopmans et al., 2010; Tian et al., 2010; Bandettini, 2012). In fact, neurovascular coupling is reported to vary by cortical depth and layer (Goense et al., 2012).

In a longitudinal study investigating CT, GM volume, CA across the lifespan, Storsve et al. (2014) reported cortical morphology metric specific and region specific rates of mean annual percentage change (APC) in healthy adults aged 23–87 years. In most regions, GM volume has a mean APC of –0.51, CT of –0.35, and CA of –0.19. Other longitudinal studies in older adults, report similar reductions: in GM volume, mean APC ranging from –0.5 to –2.1 ± 1.6% (Tang et al., 2001; Fjell et al., 2009), in CT, mean APC –0.3% (Shaw et al., 2016), in WM tracts, mean APC ranging from –0.20 to –0.65 depending on the tract location (Storsve et al., 2016). In our sample, the change in cortical morphology from scan 1 to scan 2 (2.5 years apart) was not statistically significant. However, the findings reported by these longitudinal studies suggest that while the neuroanatomical alterations may be too subtle to reach statistically significance in the investigated timespan of 2.5 years, they may still contribute to BOLD_SD_ age-related findings. Particularly, accounting for CT age-related changes may help “unmask” the functional value of BOLD_SD._

While the relationship between cortical morphology metrics in aging is dynamic, GM volume changes were largely accounted by changes in CT rather than CA, highlighting the importance of tracking changes in CT (Storsve et al., 2014). In fact, studies show that CT and CA are genetically independent (Panizzon et al., 2009). Their neurodevelopment in the lifespan is largely independent of each other suggesting that they should be considered as separate metrics with different contributions to cortical volume (Im et al., 2008; Winkler et al., 2010; Eyler et al., 2011; Lemaitre et al., 2012; Storsve et al., 2014). Furthermore, studies show that that some cortices experience decrease in cortical morphology metrics at a higher rate than others (Storsve et al., 2014).

The hemodynamic properties of each brain region are highly correlated with its cortical structure (Ulrich and Yablonskiy, 2016; Wen et al., 2018). Studies based on quantitative gradient recalled echo (qGRE) analysis use the GRE signal decay rate parameter (R2t^∗^) to gather information on tissue-specific contributions to the fMRI signal. Regional R2t^∗^ metric variations are associated with variations in neuronal density and myelination (positive correlation), as well as glial cells and synapses concentration (i.e., negative correlation) (Wen et al., 2018). In normal aging R2t^∗^ increases in all cortical regions (Zhao et al., 2016). Thicker regions show the opposite pattern, respectively, decreased neuronal density, and higher concentration of glial cells and synapses relative to neurons. Thicker areas also tend to extract less oxygen from the blood, as measured by oxygen extraction ratios (i.e., expressed as local-to-global ratio). These findings indicate that laminar differences in cellular content impact neurovascular coupling mechanisms, which in turn may compromise the power of BOLD_SD_ measurements to detect “real” changes in neuronal variability processing (Harris et al., 2011). Although, laminar differences in BOLD_SD_ remain rather elusive, our study suggests the that CT should be considered in BOLD variability studies.

### Age-Related Changes in BOLD_SD_

In our study, we did not find a significant change in BOLD_SD_, likely due to the low timespan between scans (2.5 years), and relatively low sample (*n* = 28). Overall, most studies reporting a decrease in BOLD_SD_ suggest that this finding may indicate structural reductions in synaptic complexity and integrity, as well as functional decline in neural optimization and flexibility (Garrett et al., 2010). Burzynska et al. (2015) reported a positive correlation between increased microstructural integrity of WM and BOLD_SD_ in healthy adults, consequently warranting the consideration of structural alterations in variability studies. Nomi et al. (2017) found increases in variability in bilateral anterior insula, anterior cingulate and ventral temporal cortex, and decreases in BOLD_SD_ in sensory brain regions and other subcortical areas, in participants with ages ranging from 6 to 86 years. In their study they accounted for GM volume but not CT or CA. Two seminal studies investigated BOLD_SD_ differences in aging alone (Garrett et al., 2010) and in relation to performance on cognitive tasks (Garrett et al., 2011) between a young group of participants (20–30 years) and an older one (56–85 years) and found both increases and decreases in regional variability with younger age alone, and younger age and better performance (Garrett et al., 2011) making it rather difficult to isolate specific key contributing regions. Most regions in these two studies showed the same trend but there were some inconsistencies. For example, superior frontal gyrus was reported to show greater variability with younger age and better performance (Garrett et al., 2011) in one study, while another one reported that its variability increases with age (Garrett et al., 2010). CT was not considered in any of these studies. The relationship between cognitive performance and/or flexibility and BOLD_SD_ is definitely complex and task dependent. Behavioral studies indicate age-related differences in intra-individual variability on cognitive performance tests involving reaction time, and working memory. Older adults showing higher intra-individual variability on reaction time tests than younger adults, while the opposite is observed on working memory tests (Fagot et al., 2018). Additionally, the relationship between BOLD_SD_ change, aging and cognitive health has not been a consistent finding. Some studies have found increased BOLD in healthy older adults (Baum and Beauchamp, 2014), Alzheimer’s (Scarapicchia et al., 2018), attention deficit hyperactivity disorder (ADHD) (Nomi et al., 2018), and other neurological disorders (Zöller et al., 2017).

### Other Possible Confounds in BOLD_SD_ Studies

Most studies investigating BOLD variability utilized a cross-sectional design while we used a within-subject design. This design is a highlight of our study because it allows for the investigation of normative age-related differences, specifically intra-individual effects of the processing of aging on cortical morphology and BOLD_SD_, rather than process of aging simply age differences across groups. Importantly, the within-subject design helps account for numerous sources of individual differences that may affect BOLD variability such as differences in dopaminergic neurotransmission (Guitart-Masip et al., 2016; Alavash et al., 2018), significant differences in vascular features, pain sensitivity and coping (Rogachov et al., 2016), clinical symptomology (Ke et al., 2015; Nomi et al., 2018; Zöller et al., 2018), and even differences in tendency for financial risk taking (Samanez-Larkin et al., 2010). Given that in our study the participants were scanned at an interval of approximately 2.5 years, we were able to at least partially account for such factors. Furthermore, it is clear from the literature that there are numerous environmental factors [i.e., socioeconomic status, education (Chan et al., 2018)] that may contribute to individual neuroanatomical alterations, which in turn may confound BOLD variability studies. This further supports the findings of our study, specifically that CT is a neuroanatomical metric that should be accounted for.

Lastly, there may be inconsistency in results between studies due to: (1) using task-fMRI and resting-state fMRI, (2) calculation of BOLD signal variability using standard deviation vs. mean-square successive difference) (for review, Garrett et al., 2013b), (3) type of statistical analysis performed (e.g., partial least squares method, LMM, general linear model), and (4) not accounting for contribution of CT and the other mentioned sources of individual differences.

## Study Limitations

The main limitations, as discussed above and further addressed by Garrett et al. (2013b); Scarapicchia et al. (2018), are the lack of standardization in acquiring and analyzing the fMRI data. Additionally, the sample used in our study consists of a relatively small sample of older adults and rather short scan-rescan time, consequently this limits our ability to make strong generalization to other age groups or longer time spans, respectively. Nevertheless, since studies show that the brain undergoes extensive annual structural alterations across the lifespan at different rates, our finding that CT contributes to BOLD_SD_ alterations, remains an important consideration.

## Conclusion

In conclusion, contrary to a view that BOLD variability is just “noise,” we consider it to be emerging as an important metric of normal aging. Keeping in mind that across the lifespan there are considerable cortical morphometry alterations and that cortical depth affects the BOLD signal, we reported that CT contributes to BOL_DSD_ changes, in an older sample of health adults. An increasing number of studies are considering healthy regional BOLD_SD_ changes in the context of functional networks (Rogachov et al., 2016; Nomi et al., 2017). Pursuing this direction while accounting for CT and other possible confounding factors such as dopaminergic neurotransmission, socioeconomic background etc., should reveal new insights into the mechanisms behind age-related neural processes.

## Data Availability

All datasets generated for this study are included in the manuscript and/or the supplementary files.

## Author Contributions

DP, RE, and SR conceived and designed this specific project on the data collected by NM and AR. DP analyzed the data. All co-authors participated in writing of the manuscript.

## Conflict of Interest Statement

The authors declare that the research was conducted in the absence of any commercial or financial relationships that could be construed as a potential conflict of interest.

## Footnotes

1 http://surfer.nmr.mgh.harvard.edu/

2 www.R-project.org

## References

[B1] Achiron A. Chapman J. Tal S. Bercovich E. Gil H. Achiron A. (2013). Superior temporal gyrus thickness correlates with cognitive performance in multiple sclerosis. *Brain Struct. Funct.* 218 943–950. 10.1007/s00429-012-0440-3 22790785

[B2] Alavash M. Lim S. J. Thiel C. Sehm B. Deserno L. Obleser J. (2018). Dopaminergic modulation of hemodynamic signal variability and the functional connectome during cognitive performance. *Neuroimage* 172 341–356. 10.1016/j.neuroimage.2018.01.048 29410219

[B3] Armbruster-Genc D. J. N. Ueltzhoffer K. Fiebach C. J. (2016). Brain signal variability differentially affects cognitive flexibility and cognitive stability. *J. Neurosci.* 36 3978–3987. 10.1523/JNEUROSCI.2517-14.2016 27053205 PMC6705511

[B4] Bandettini P. A. (2012). The BOLD plot thickens: sign- and layer-dependent hemodynamic changes with activation. *Neuron* 76 468–469. 10.1016/j.neuron.2012.10.026 23141059

[B5] Bates D. (2005). Fitting linear mixed models in R. *R News* 5 27–30. 10.1159/000323281 21196786 PMC6515857

[B6] Baum S. H. Beauchamp M. S. (2014). Greater BOLD variability in older compared with younger adults during audiovisual speech perception. *PLoS One* 9:e111121. 10.1371/journal.pone.0111121 25337918 PMC4206517

[B7] Beckmann C. F. Smith S. M. (2004). Probabilistic independent component analysis for functional magnetic resonance imaging. *IEEE Trans. Med. Imaging* 23 137–152. 10.1109/TMI.2003.822821 14964560

[B8] Benjamini Y. Yekutieli D. (2001). The control of the false discovery rate in multiple testing under dependency. *Ann. Stat.* 29 1165–1188. 10.1214/aos/1013699998 18298808

[B9] Bernal-Rusiel J. L. Reuter M. Greve D. N. Fischl B. Sabuncu M. R. (2013). Spatiotemporal linear mixed effects modeling for the mass-univariate analysis of longitudinal neuroimage data. *Neuroimage* 81 358–370. 10.1016/j.neuroimage.2013.05.049 23702413 PMC3816382

[B10] Burzynska A. Z. Wong C. N. Voss M. W. Cooke G. E. McAuley E. Kramer A. F. (2015). White matter integrity supports BOLD signal variability and cognitive performance in the aging human brain. *PLoS One* 10:e0120315. 10.1371/journal.pone.0120315 25853882 PMC4390282

[B11] Chan M. Y. Na J. Agres P. F. Savalia N. K. Park D. C. Wig G. S. (2018). Socioeconomic status moderates age-related differences in the brain’s functional network organization and anatomy across the adult lifespan. *Proc. Natl. Acad. Sci. U.S.A.* 115 E5144–E5153. 10.1073/pnas.1714021115 29760066 PMC5984486

[B12] Dale A. M. Fischl B. Sereno M. I. (1999). Cortical surface-based analysis. I. segmentation and surface reconstruction. *Neuroimage* 9 179–194. 10.1006/nimg.1998.0395 9931268

[B13] Destrieux C. Fischl B. Dale A. Halgren E. (2010). Automatic parcellation of human cortical gyri and sulci using standard anatomical nomenclature. *Neuroimage* 53 1–15. 10.1016/j.neuroimage.2010.06.010 20547229 PMC2937159

[B14] Dotson V. M. Szymkowicz S. M. Sozda C. N. Kirton J. W. Green M. L. O’Shea A. (2016). Age differences in prefrontal surface area and thickness in middle aged to older adults. *Front. Aging Neurosci.* 7:250 10.3389/fnagi.2015.00250 PMC4717301 26834623

[B15] Driscoll I. Davatzikos C. An Y. Wu X. Shen D. Kraut M. (2009). Longitudinal pattern of regional brain volume change differentiates normal aging from MCI. *Neurology* 72 1906–1913. 10.1212/WNL.0b013e3181a82634 19487648 PMC2690968

[B16] Eyler L. T. Prom-Wormley E. Panizzon M. S. Kaup A. R. Fennema-Notestine C. Neale M. C. (2011). Genetic and environmental contributions to regional cortical surface area in humans: a magnetic resonance imaging twin study. *Cereb. Cortex* 21 2313–2321. 10.1093/cercor/bhr013 21378112 PMC3169660

[B17] Fagot D. Mella N. Borella E. Ghisletta P. Lecerf T. de Ribaupierre A. (2018). Intra-individual variability from a lifespan perspective: a comparison of latency and accuracy measures. *J. Intell.* 6 1–18. 10.3390/jintelligence6010016 PMC6480759 31162443

[B18] Fischl B. Dale A. M. (2000). Measuring the thickness of the human cerebral cortex from magnetic resonance images. *Proc. Natl. Acad. Sci. U.S.A.* 97 11050–11055. 10.1073/pnas.200033797 10984517 PMC27146

[B19] Fischl B. Liu A. Dale A. M. (2001). Automated manifold surgery: constructing geometrically accurate and topologically correct models of the human cerebral cortex. *IEEE Trans. Med. Imaging* 20 70–80. 10.1109/42.906426 11293693

[B20] Fischl B. Sereno M. I. Dale A. M. (1999a). Cortical surface-based analysis. II: inflation, flattening, and a surface-based coordinate system. *Neuroimage* 9 195–207. 10.1006/nimg.1998.0396 9931269

[B21] Fischl B. Sereno M. I. Tootell R. B. Dale A. M. (1999b). High-resolution intersubject averaging and a coordinate system for the cortical surface. *Hum. Brain Mapp.* 8 272–284. 10619420 10.1002/(SICI)1097-0193(1999)8:4<272::AID-HBM10>3.0.CO;2-4 PMC6873338

[B22] Fjell A. M. Walhovd K. B. Fennema-Notestine C. McEvoy L. K. Hagler D. J. Holland D. (2009). One-year brain atrophy evident in healthy aging. *J. Neurosci.* 29 15223–15231. 10.1523/JNEUROSCI.3252-09.2009 19955375 PMC2827793

[B23] Fjell A. M. Walhovd K. B. Reinvang I. Lundervold A. Salat D. Quinn B. T. (2006). Selective increase of cortical thickness in high-performing elderly - structural indices of optimal cognitive aging. *Neuroimage* 29 984–994. 10.1016/j.neuroimage.2005.08.007 16176876

[B24] Garrett D. D. Kovacevic N. McIntosh A. R. Grady C. L. (2010). Blood oxygen level-dependent signal variability is more than just noise. *J. Neurosci.* 30 4914–4921. 10.1523/JNEUROSCI.5166-09.2010 20371811 PMC6632804

[B25] Garrett D. D. Kovacevic N. McIntosh A. R. Grady C. L. (2011). The importance of being variable. *J. Neurosci.* 31 4496–4503. 10.1523/JNEUROSCI.5641-10.2011 21430150 PMC3104038

[B26] Garrett D. D. Kovacevic N. McIntosh A. R. Grady C. L. (2013a). The modulation of BOLD variability between cognitive states varies by age and processing speed. *Cereb. Cortex* 23 684–693. 10.1093/cercor/bhs055 22419679 PMC3823571

[B27] Garrett D. D. Samanez-Larkin G. R. MacDonald S. W. S. Lindenberger U. McIntosh A. R. Grady C. L. (2013b). Moment-to-moment brain signal variability: a next frontier in human brain mapping? *Neurosci. Biobehav. Rev.* 37 610–624. 10.1016/j.neubiorev.2013.02.015 23458776 PMC3732213

[B28] Garrett D. D. Lindenberger U. Hoge R. D. Gauthier C. J. (2017). Age differences in brain signal variability are robust to multiple vascular controls. *Sci. Rep.* 7:10149. 10.1038/s41598-017-09752-7 28860455 PMC5579254

[B29] Garrett D. D. McIntosh A. R. Grady C. L. (2014). Brain signal variability is parametrically modifiable. *Cereb. Cortex* 24 2931–2940. 10.1093/cercor/bht150 23749875 PMC4193462

[B30] Goense J. Merkle H. Logothetis N. K. (2012). High-resolution FMRI reveals laminar differences in neurovascular coupling between positive and negative BOLD responses. *Neuron* 76 629–639. 10.1016/j.neuron.2012.09.019 23141073 PMC5234326

[B31] Grady C. L. Garrett D. D. (2014). Understanding variability in the BOLD signal and why it matters for aging. *Brain Imaging Behav.* 8 274–283. 10.1007/s11682-013-9253-0 24008589 PMC3922711

[B32] Guitart-Masip M. Salami A. Garrett D. Rieckmann A. Lindenberger U. Bäckman L. (2016). BOLD variability is related to dopaminergic neurotransmission and cognitive aging. *Cereb. Cortex* 26 2074–2083. 10.1093/cercor/bhv029 25750252

[B33] Harris J. J. Reynell C. Attwell D. (2011). The physiology of developmental changes in BOLD functional imaging signals. *Dev. Cogn. Neurosci.* 1 199–216. 10.1016/j.dcn.2011.04.001 22436508 PMC6987565

[B34] Hogstrom L. J. Westlye L. T. Walhovd K. B. Fjell A. M. (2013). The structure of the cerebral cortex across adult life: age-related patterns of surface area, thickness, and gyrification. *Cereb. Cortex* 23 2521–2530. 10.1093/cercor/bhs231 22892423

[B35] Huber L. Goense J. Kennerley A. J. Trampel R. Guidi M. Reimer E. (2015). Cortical lamina-dependent blood volume changes in human brain at 7 T. *Neuroimage* 107 23–33. 10.1016/j.neuroimage.2014.11.046 25479018

[B36] Im K. Lee J. M. Lyttelton O. Kim S. H. Evans A. C. Kim S. I. (2008). Brain size and cortical structure in the adult human brain. *Cereb. Cortex* 18 2181–2191. 10.1093/cercor/bhm244 18234686

[B37] Jenkinson M. Beckmann C. F. Behrens T. E. J. Woolrich M. W. Smith S. M. (2012). FSL. *Neuroimage* 62 782–790. 10.1016/j.neuroimage.2011.09.015 21979382

[B38] Jiang J. Sachdev P. Lipnicki D. M. Zhang H. Liu T. Zhu W. (2014). A longitudinal study of brain atrophy over two years in community-dwelling older individuals. *Neuroimage* 86 203–211. 10.1016/j.neuroimage.2013.08.022 23959201

[B39] Ke J. Zhang L. Qi R. Xu Q. Li W. Hou C. (2015). Altered blood oxygen level-dependent signal variability in chronic post-traumatic stress disorder during symptom provocation. *Neuropsychiatr. Dis. Treat.* 11 1805–1815. 10.2147/NDT.S87332 26229476 PMC4517522

[B40] Kielar A. Deschamps T. Chu R. K. Jokel R. Khatamian Y. B. Chen J. J. (2016). Identifying dysfunctional cortex: dissociable effects of stroke and aging on resting state dynamics in MEG and fMRI. *Front. Aging Neurosci.* 8:40. 10.3389/fnagi.2016.00040 26973515 PMC4776400

[B41] Koopmans P. J. Barth M. Norris D. G. (2010). Layer-specific BOLD activation in human V1. *Hum. Brain Mapp.* 31 1297–1304. 10.1002/hbm.20936 20082333 PMC6870878

[B42] Kuznetsova A. Brockhoff P. B. Christensen R. H. B. (2017). LmerTest package: tests in linear mixed effects models. *J. Stat. Softw.* 82 1–26. 10.18637/jss.v082.i13

[B43] Lemaitre H. Goldman A. L. Sambataro F. Verchinski B. A. Meyer-Lindenberg A. Weinberger D. R. (2012). Normal age-related brain morphometric changes: nonuniformity across cortical thickness, surface area and gray matter volume? *Neurobiol. Aging* 33 617.e1–617.e9. 10.1016/j.neurobiolaging.2010.07.013 20739099 PMC3026893

[B44] Makedonov I. Black S. E. MacIntosh B. J. (2013). BOLD FMRI in the white matter as a marker of aging and small vessel disease. *PLoS One* 8:e67652. 10.1371/journal.pone.0067652 23844047 PMC3699638

[B45] Makedonov I. Chen J. J. Masellis M. MacIntosh B. J. (2016). Physiological fluctuations in white matter are increased in Alzheimer’s disease and correlate with neuroimaging and cognitive biomarkers. *Neurobiol. Aging* 37 12–18. 10.1016/j.neurobiolaging.2015.09.010 26476600

[B46] McGinnis S. M. Brickhouse M. Pascual B. Dickerson B. C. (2011). Age-related changes in the thickness of cortical zones in humans. *Brain Topogr.* 24 279–291. 10.1007/s10548-011-0198-6 21842406 PMC3600370

[B47] Mella N. de Ribaupierre S. Eagleson R. de Ribaupierre A. (2013). Cognitive intraindividual variability and white matter integrity in aging. *ScientificWorldJournal* 2013:350623. 10.1155/2013/350623 24174913 PMC3794509

[B48] Nomi J. S. Bolt T. S. Chiemeka Ezie C. E. Uddin L. Q. Heller A. S. (2017). Moment-to-moment BOLD signal variability reflects regional changes in neural flexibility across the lifespan. *J. Neurosci.* 37 5539–5548. 10.1523/JNEUROSCI.3408-16.2017 28473644 PMC5452342

[B49] Nomi J. S. Schettini E. Voorhies W. Bolt T. S. Heller A. S. Uddin L. Q. (2018). Resting-state brain signal variability in prefrontal cortex is associated with ADHD symptom severity in children. *Front. Hum. Neurosci.* 12:90. 10.3389/fnhum.2018.00090 29593515 PMC5857584

[B50] Pacheco J. Goh J. O. Kraut M. A. Ferrucci L. Resnick S. M. (2015). Greater cortical thinning in normal older adults predicts later cognitive impairment. *Neurobiol. Aging* 36 903–908. 10.1016/j.neurobiolaging.2014.08.031 25311277 PMC4315712

[B51] Panizzon M. S. Fennema-Notestine C. Eyler L. T. Jernigan T. L. Prom-Wormley E. Neale M. (2009). Distinct genetic influences on cortical surface area and cortical thickness. *Cereb. Cortex* 19 2728–2735. 10.1093/cercor/bhp026 19299253 PMC2758684

[B52] Petracca M. Saiote C. Bender H. A. Arias F. Farrell C. Magioncalda P. (2017). Synchronization and variability imbalance underlie cognitive impairment in primary-progressive multiple sclerosis. *Sci. Rep.* 7:46411. 10.1038/srep46411 28429774 PMC5399449

[B53] R Core Team (2013). *R: A Language and Environment for Statistical Computing.* Vienna: R Foundation for Statistical Computing Available at: https://www.R-project.org/

[B54] Raz N. (2005). Regional brain changes in aging healthy adults: general trends, individual differences and modifiers. *Cereb. Cortex* 15 1676–1689. 10.1093/cercor/bhi044 15703252

[B55] Reuter M. Rosas H. D. Fischl B. (2010). Highly accurate inverse consistent registration: a robust approach. *Neuroimage* 53 1181–1196. 10.1016/j.neuroimage.2010.07.020 20637289 PMC2946852

[B56] Rogachov A. Cheng J. C. Erpelding N. Hemington K. S. Crawley A. P. Davis K. D. (2016). Regional brain signal variability: a novel indicator of pain sensitivity and coping. *Pain* 157 2483–2492. 10.1097/j.pain.0000000000000665 27429176

[B57] Rossini P. M. Rossi S. Babiloni C. Polich J. (2007). Clinical neurophysiology of aging brain: from normal aging to neurodegeneration. *Prog. Neurobiol.* 83 375–400. 10.1016/j.pneurobio.2007.07.010 17870229

[B58] Salat D. H. Buckner R. L. Snyder A. Z. Greve D. N. Desikan R. S. R. Busa E. (2004). Thinning of the cerebral cortex in aging. *Cereb. Cortex* 14 721–730. 10.1093/cercor/bhh032 15054051

[B59] Salat D. H. Lee S. Y. van der Kouwe A. J. Greve D. N. Fischl B. Rosas H. D. (2009). Age-associated alterations in cortical gray and white matter signal intensity and gray to white matter contrast. *Neuroimage* 48 21–28. 10.1016/j.neuroimage.2009.06.074 19580876 PMC2750073

[B60] Samanez-Larkin G. R. Kuhnen C. M. Yoo D. J. Knutson B. (2010). Variability in nucleus accumbens activity mediates age-related suboptimal financial risk taking. *J. Neurosci.* 30:1426. 10.1523/JNEUROSCI.4902-09.2010 20107069 PMC2821055

[B61] Scarapicchia V. Mazerolle E. L. Fisk J. D. Ritchie L. J. Gawryluk J. R. (2018). Resting state BOLD variability in Alzheimer’s disease: a marker of cognitive decline or cerebrovascular status? *Front. Aging Neurosci.* 10:39 10.3389/fnagi.2018.00039 PMC5826397 29515434

[B62] Ségonne F. Dale A. M. Busa E. Glessner M. Salat D. Hahn H. K. (2004). A hybrid approach to the skull stripping problem in MRI. *Neuroimage* 22 1060–1075. 10.1016/j.neuroimage.2004.03.032 15219578

[B63] Segonne F. Pacheco J. Fischl B. (2007). Geometrically accurate topology-correction of cortical surfaces using nonseparating loops. *IEEE Trans. Med. Imaging* 26 518–529. 10.1109/TMI.2006.887364 17427739

[B64] Shaw M. E. Sachdev P. S. Anstey K. J. Cherbuin N. (2016). Age-related cortical thinning in cognitively healthy individuals in their 60s: the path through life study. *Neurobiol. Aging* 39 202–209. 10.1016/j.neurobiolaging.2015.12.009 26923417

[B65] Singh V. Chertkow H. Lerch J. P. Evans A. C. Dorr A. E. Kabani N. J. (2006). Spatial patterns of cortical thinning in mild cognitive impairment and Alzheimer’s disease. *Brain* 129(Pt 11), 2885–2893. 10.1093/brain/awl256 17008332

[B66] Sled J. G. Zijdenbos A. P. Evans A. C. (1998). A nonparametric method for automatic correction of intensity nonuniformity in MRI data. *IEEE Trans. Med. Imaging* 17 87–97. 10.1109/42.668698 9617910

[B67] Smith S. M. (2002). Fast robust automated brain extraction. *Hum. Brain Mapp.* 17 143–155. 10.1002/hbm.10062 12391568 PMC6871816

[B68] Smith S. M. Jenkinson M. Woolrich M. W. Beckmann C. F. Behrens T. E. J. Johansen-Berg H. (2004). Advances in functional and structural MR image analysis and implementation as FSL. *Neuroimage* 23(Suppl. 1), S208–S219. 10.1016/j.neuroimage.2004.07.051 15501092

[B69] Storsve A. B. Fjell A. M. Tamnes C. K. Westlye L. T. Overbye K. Aasland H. W. (2014). Differential longitudinal changes in cortical thickness, surface area and volume across the adult life span: regions of accelerating and decelerating change. *J. Neurosci.* 34 8488–8498. 10.1523/JNEUROSCI.0391-14.2014 24948804 PMC6608217

[B70] Storsve A. B. Fjell A. M. Yendiki A. Walhovd K. B. (2016). Longitudinal changes in white matter tract integrity across the adult lifespan and its relation to cortical thinning. *PLoS One* 11:e0156770. 10.1371/journal.pone.0156770 27253393 PMC4890742

[B71] Tang Y. Whitman G. T. Lopez I. Baloh R. W. (2001). Brain volume changes on longitudinal magnetic resonance imaging in normal older people. *J. Neuroimaging* 11 393–400. 10.1111/j.1552-6569.2001.tb00068.x 11677879

[B72] Thambisetty M. Wan J. Carass A. An Y. Prince J. L. Resnick S. M. (2010). Longitudinal changes in cortical thickness associated with normal aging. *Neuroimage* 52 1215–1223. 10.1016/j.neuroimage.2010.04.258 20441796 PMC2910226

[B73] Tian P. Teng I. C. May L. D. Kurz R. Lu K. Scadeng M. (2010). Cortical depth-specific microvascular dilation underlies laminar differences in blood oxygenation level-dependent functional MRI signal. *Proc. Natl. Acad. Sci. U.S.A.* 107 15246–15251. 10.1073/pnas.1006735107 20696904 PMC2930564

[B74] Ulrich X. Yablonskiy D. A. (2016). Separation of cellular and BOLD contributions to T2^∗^ signal relaxation. *Magn. Reson. Med.* 75 606–615. 10.1002/mrm.25610 25754288 PMC4565789

[B75] Wen J. Goyal M. S. Astafiev S. V. Raichle M. E. Yablonskiy D. A. (2018). Genetically defined cellular correlates of the baseline brain MRI signal. *Proc. Natl. Acad. Sci. U.S.A.* 115 E9727–E9736. 10.1073/pnas.1808121115 30254176 PMC6187202

[B76] Winkler A. M. Kochunov P. Blangero J. Almasy L. Zilles K. Fox P. T. (2010). Cortical thickness or grey matter volume? The importance of selecting the phenotype for imaging genetics studies. *Neuroimage* 53 1135–1146. 10.1016/j.neuroimage.2009.12.028 20006715 PMC2891595

[B77] Winkler A. M. Sabuncu M. R. Yeo B. T. T. Fischl B. Greve D. N. Kochunov P. (2012). Measuring and comparing brain cortical surface area and other areal quantities. *Neuroimage* 61 1428–1443. 10.1016/j.neuroimage.2012.03.026 22446492 PMC3641659

[B78] Zhang Y. Brady M. Smith S. (2001). Segmentation of brain MR images through a hidden markov random field model and the expectation-maximization algorithm. *IEEE Trans. Med. Imaging* 20 45–57. 10.1109/42.906424 11293691

[B79] Zhao Y. Wen J. Cross A. H. Yablonskiy D. A. (2016). On the relationship between cellular and hemodynamic properties of the human brain cortex throughout adult lifespan. *Neuroimage* 133 417–429. 10.1016/j.neuroimage.2016.03.022 26997360 PMC4889562

[B80] Ziegler D. A. Piguet O. Salat D. H. Prince K. Connally E. Corkin S. (2010). Cognition in healthy aging is related to regional white matter integrity, but not cortical thickness. *Neurobiol. Aging* 31 1912–1926. 10.1016/j.neurobiolaging.2008.10.015 19091444 PMC2996721

[B81] Zöller D. Padula M. C. Sandini C. Schneider M. Scariati E. Van De Ville D. (2018). Psychotic symptoms influence the development of anterior cingulate BOLD variability in 22q11.2 deletion syndrome. *Schizophr. Res.* 193 319–328. 10.1016/j.schres.2017.08.003 28803847

[B82] Zöller D. Schaer M. Scariati M. Padula C. M. Eliez S. Van De Ville D. (2017). Disentangling resting-state BOLD variability and PCC functional connectivity in 22q11.2 deletion syndrome. *Neuroimage* 149 85–97. 10.1016/j.neuroimage.2017.01.064 28143774

[B83] Zuur A. F. Leno E. N. Walker N. J. Saveliev A. A. Smith G. M. (2011). *Mixed Effects Models and Extensions in Ecology with R.* New York, NY: Springer.

